# New insights into the epitranscriptomic control of pluripotent stem cell fate

**DOI:** 10.1038/s12276-022-00824-x

**Published:** 2022-10-21

**Authors:** Young Hyun Che, Hojae Lee, Yong Jun Kim

**Affiliations:** 1 Department of Biomedical Science, Graduate School, Kyung Hee University, Seoul, 02447 Korea; 2 Cedars-Sinai Medical Center, Biomanufacturing Center, Los Angeles, CA 90069 USA; 3 Department of Pathology, College of Medicine, Kyung Hee University, Seoul, 02447 Korea; 4 KHU-KIST Department of Converging Science and Technology, Kyung Hee University, Seoul, 02447 Korea

**Keywords:** Embryonic stem cells, Transcriptomics

## Abstract

Each cell in the human body has a distinguishable fate. Pluripotent stem cells are challenged with a myriad of lineage differentiation options. Defects are more likely to be fatal to stem cells than to somatic cells due to the broad impact of the former on early development. Hence, a detailed understanding of the mechanisms that determine the fate of stem cells is needed. The mechanisms by which human pluripotent stem cells, although not fully equipped with complex chromatin structures or epigenetic regulatory mechanisms, accurately control gene expression and are important to the stem cell field. In this review, we examine the events driving pluripotent stem cell fate and the underlying changes in gene expression during early development. In addition, we highlight the role played by the epitranscriptome in the regulation of gene expression that is necessary for each fate-related event.

## Epigenetics: chemical tags on RNA mediate stem cell fate

Chemical modifications made to key gene transcripts, messenger RNAs carrying genetic information from DNA, help to govern the differentiation potential and developmental stage of stem cells in the early embryo. Yong Jun Kim of Kyung Hee University in Seoul, South Korea, and colleagues review the molecular pathways that control the identity of stem cells and highlight how these epigenetic changes during embryonic development can impact the fate of cells. The addition of methyl groups to particular parts of gene transcripts that encode for regulatory proteins seems to be an especially common mechanism for maintaining pluripotency, the ability to form any cell type in the body. Other types of modification also play a role, collectively impacting stem cell dynamics in ways that could be important for regenerative medicine.

## Introduction

Stem cells exhibit the potential to differentiate into various cell types^1^. Therefore, stem cells have been useful in biological and medical research^2^. Stem cells have the advantage of inducibility to obtain and utilize the cell types suitable for a particular research purpose. In particular, stem cell types affect different developmental stages and differentiation lineages^3,4^. The usefulness of stem cells is based primarily on their ability to undergo various cell development stages, allowing researchers to obtain specific cell types with phenotypes at the appropriate developmental time based on the stage of acquisition^5^. Using lineage differentiated stem cells has enabled research model establishment for the direct investigation of molecular mechanisms, phenotypes, and therapeutic strategies in phenotype-relevant human cells^6–8^. Stem cells in the early stages of development exhibit a wide range of differentiation properties^2^. For example, zygote cells proliferate to form a morula, a group of stem cells with the same multicellular differentiation potential, including embryonic cells that form an organism and supporting cells needed to maintain proper embryonic development^9^. This broad differentiation potential and totipotency of stem cells gradually decrease as stem cells proliferate and acquire the necessary phenotypes during development, eventually progressing to a state called pluripotency^10^. Embryonic stem cells (ESCs) extracted from the inner cell mass (ICM) of a blastocyst are stem cells in the pluripotent state^11^. Although these cells have lost the ability to differentiate into cells that form extraembryonic tissues, such as the placenta and yolk sac, they retain the ability to differentiate into cells that can form fetal tissues and organs^12^. Thus, human ESCs (hESCs) in a pluripotent state are actively employed in developmental and pathological research that integrates aspects of human body pathophysiology^13,14^. However, both the totipotent and pluripotent states of cells are dynamic, and the range of differentiation potential changes gradually with the progressive maturation of stem cells^15^. Furthermore, whereas totipotent stem cells are characterized by their homogenous proliferation, human pluripotent stem cells (hPSCs) gradually show cellular diversity in the ICM of a blastocyst^16,17^. The pluripotency of stem cells is thus limited, leading to the generation of three heterogeneous germ populations through pattern and structural formation events and gastrulation^18^. Thus, understanding the biological events and molecular mechanisms that control the properties of hPSCs is critical. However, only a subset of the molecular pathways governing stem cell pluripotency is understood.

The state of stem cells is determined by the influence of spatiotemporal location and ambient environmental conditions that change on the basis of developmental processes^19^. In a proliferating mass of stem cells, the topological information of each cell leads to diverse cell polarity and plasticity^20,21^. Both the matrix surrounding stem cells and various environmentally induced signaling pathways trigger alterations in stem cell activity and behavior^22^. Through the control of gene expression, these factors eventually lead to biological changes, functioning as intrinsic fate regulators operated by the stem cells themselves^23,24^. Gene expression patterns regulate the biological properties of cells and define cell types^25^. Similar to those of somatic cells, the functions of stem cells are controlled by the molecular actions of proteins encoded by cell-type-specific genes^26^. Gene expression control, therefore, is a key factor in determining the state and function of stem cells, regardless of intrinsic or extrinsic triggers^24,27^. To ensure the timely and accurate regulation of required gene expression, stem cells engage well-established transcription machineries and epigenetic regulatory systems^28^. For instance, recent studies have suggested that an epitranscriptomic gene regulatory system changes the protein translation efficiency and persistence of messenger RNA (mRNA) through chemical modifications of transcribed mRNAs^29,30^. Compared with somatic cells, hPSCs have immature genetic and epigenetic regulatory mechanisms^31,32^, which can affect precise gene expression control, resulting in alterations in protein expression patterns that can be fatal^33^. Nonetheless, PSCs in the early stages of development preserve an accurate fate decision process based on tight gene expression control^34^. In addition to conventional gene regulation, other molecular mechanisms may play a role in the control of gene expression in PSCs.

In this review, we summarize changes in the state of PSCs during the early stages of development and the regulatory mechanisms underlying these changes. Additionally, we discuss recent research on gene regulation at the transcriptome level in relation to the regulation of PSC fate. Although a number of studies have reported distinct properties of hPSCs compared with mouse PSCs due to interspecies differences, the use of mouse embryos can provide sufficient knowledge for understanding human embryonic development. Here, we present studies on both mice and human cells and point out whether the results have been derived from human or animal models.

## State of pluripotent stem cells (PSCs) during early development

**Fig. 1:**
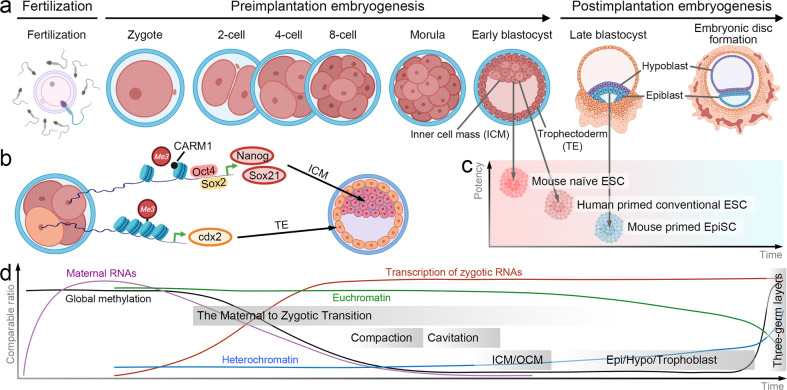
Dynamics of stem cell development. **a** Morphological changes of stem cells according to the stage of stem cell development. **b** Molecular mechanism in the acquisition of heterogeneity. **c** Pluripotent state of stem cells derived during different developmental stages. **d** Molecular characteristics during stem cell development.

Stem-cell development generates diverse groups of cells with different properties via the generation of identical multiple-twin cells from a starting single cell (Fig. 1a)^35–37^. Immediately after fertilization, zygote cells continue to divide, forming a group of 16-homogenous cells that comprise the early morula^38^. As this cell group continues to divide, differences in environmental conditions, including topographical forces applied to each cell in the mass, are created. These differences result in the generation of the first heterogeneous population: the ICM and trophectoderm (TE, Fig. 1b)^35,39,40^. Each cell group constituting the preimplantation blastocyst exhibits distinct differentiation potential for the production of nonoverlapping progeny. Trophoblasts organize surrounding structures such as the chorion, which supports embryogenesis, whereas the ICM is critical for the formation of the embryo^41^. After implantation of a blastocyst in the maternal endometrial epithelium, the ICM undergoes subsequent morphogenetic changes. The ICM of a postimplantation blastocyst contains epiblasts and hypoblasts (Fig. 1a)^42^. The morphogenetic events include the polarization of the epiblast, which forms the central lumen that develops into the amniotic cavity; creation of the amniotic epithelium, which forms the amniotic sac membrane; and differentiation of primordial germ cells, which are precursors of eggs or sperm^43–45^. Moreover, extraembryonic mesenchyme cells derived from the hypoblast surround the generated structure to isolate it from the outer cell membrane (OCM) formed by the trophoblast^37,43,46^. Thereafter, epiblasts in the ICM form a primitive streak, gastrulate and differentiate into three germ layers: The ectoderm, mesoderm, and endoderm^47^.

HESC cultures have been established by the in vitro cultivation of cells isolated from a morula, an entire blastocyst, or an ICM^11,48,49^. Morula-derived hESCs are thought to remain in a naive state, showing unbiased differentiation potential, including the generation of trophoblasts^50^, whereas blastocyst- or ICM-derived cells are considered to exhibit a primed-like state, retaining comparably late-stage stem cell characteristics (Fig. 1c)^15^. Despite differences in cell properties, both types of hESCs exhibit the developmental spectrum that enables the generation of all somatic and germline cells^51,52^. In contrast, murine naive and primed ESCs have shown to exhibit clear differences in the molecular and cellular properties^53,54^. Previously, murine ESCs were believed to show better differentiation plasticity than human ESCs, but the limited pluripotent state epiblast stem cells (EpiSCs) was revealed. In contrast to mouse ESCs, known to be in the naive state, mouse EpiSCs in the primed state clearly show not only differences in response to activation by external signals and a decrease in self-renewal capacity but also a massive reduction in whole-animal generation efficiency because of a tetraploid composition^55,56^. Although the definition of the naive and primed state of hPSCs remains ambiguous, evidence of s naive state of mammalian stem cells, including that of primates, has been reported, suggesting the need to understand the conversion of hPSCs to the pluripotent state^57,58^.

## Mechanisms that control PSC fate

The state of PSCs, including their development and differentiation, is controlled by both intrinsic and extrinsic conditions. Deciphering the complicated signaling pathways governing the pluripotent state of stem cells is challenging, as multiple biological events, such as maturation, fate determination, and differentiation, concurrently affect stem cell development. In this context, the biological events regulating the pluripotent state of stem cells are categorized into two processes: “acquisition of heterogeneity from a homogeneous population” and “conversion from a naive to a primed state”. Each process is accompanied by an explanation of the mandating molecular pathways.

### Acquisition of heterogeneity in a homogeneous population

Among the major questions in stem cell biology, when and how is the control of clonal cells within a homogeneous population changed to produce cells with distinct and orderly fates? To form a highly organized body from an embryo comprised of symmetrical cells, the embryonic symmetry must be disrupted^59^. The importance of acquiring asymmetric fate during gastrulation to enable balanced development of the human body has been investigated in previous studies^60,61^. Complex signaling at the cellular level, as well as geometrical and topological transformation at the embryonic level, are necessary for asymmetry^62,63^. Specifically, the process that ultimately disrupts embryonic radial symmetry and triggers anteroposterior axis specification, has been found to be driven by a morphogen signaling gradient^60^. Notably, the first disruption to the symmetric organization of human cells occurs much earlier than previously thought: during the division of stem cells in the totipotent state. The emergence of the TE in blastocyst-stage embryos is the first morphological variation during embryogenesis. TE and primitive endoderm lineages generate the placenta and yolk sac extraembryonic cells, whereas cells inside the blastocyst form the ICM, the cells of which eventually divide to form the primitive endoderm and pluripotent epiblast cells. According to the most recent understanding, each cell in the early embryonic state retains identical developmental potential. Therefore, in the absence of stimulants such as morphogens, cell fate has been assumed to be determined randomly, making the existence of initiating factors driving cellular heterogeneity enigmatic. Based on single-cell transcriptome analyses, recent studies with PSCs and mouse embryos have suggested another possibility^64–67^. Differences were detected in the totipotency between cells constituting murine or human blastomeres, with each cell in the 16-cell stage exhibiting a transcriptome that differs from that in the 2-cell stage immediately after the first cleavage^66^. The gradual increase in the differential tendency of genes to contribute to lineage specification suggests that the symmetry is disrupted earlier than traditionally thought; that is, it distinguishes trophoblasts from embryoblasts after the morula stage. This finding was consistent with the results observed in several studies suggesting that only one of the cells separated in the 2-cell stage can develop into a mouse^64,68–71^.

The molecular mechanism causing this initial heterogeneity has not yet been clearly elucidated. A recent study reported that individual cells in the 4-cell-stage blastomere exhibit differential expression of epigenetic modifiers, such as that of the histone methyltransferase CARM1 (Fig. 1b)^72,73^. Differences in the expression of CARM1 regulate the DNA-binding ability of the pluripotency transcription factors SOX2 and OCT4, with activity that is affected by histone H3 arginine 26 dimethylation (H3R26me2)^74,75^. A differential increase in the expression of genes downstream of SOX2, such as *Sox21*, at the 4-cell stage has been reported to suppress the expression level of *Cdx2*^76^, a lineage specifier for TE differentiation in the 8-cell stage^77,78^. Subsequently, the reduced expression of *Cdx2* results in the decreased expression of polarity markers that define the apical domain, in turn leading to TE differentiation. Interestingly, *Cdx2* mRNA has been demonstrated to be asymmetrically localized to the apical pole in the 8-cell stage, when the embryo is undergoing apical-basal polarization^79,80^. Due to the apical localization of *Cdx2* transcripts, differential inheritance during subsequent division to the 16-cell stage might result in cells on the outside of the apical domain possessing more *Cdx2* transcripts than inner cells. Therefore, the determination of timing of these differences in gene expression between cells is important. The mechanisms underlying the determination of early mammalian stem cell fate remain unclear, largely because whether the first bifurcation in the cell fate sequence is a random event in mammalian embryo during the morula stage or is based on molecular features of differentiation that emerge prior to morphological changes remains unlcear^75,81–84^. Depending on the true pathway, either the role played by an intrinsic gene regulatory mechanism or the cellular environment condition will be predominant. An analysis of the transcriptome in multiple blastomeres revealed that after the first cleavage each cell exhibited different gene expression patterns from the pattern evident in the 2-cell stage^66^. Although evidence supports an autonomous molecular cascade for the regulation of cell fate that directs differences between cells in blastomeres early in embryonic development, the mechanisms that control precise gene expression in the open chromatin structure of early stem cells remain unclear.

### Cell conversion from a naive to a primed state

The differentiation capacity of stem cells refers to the ability to generate a range of differentiated cells, which is the basis of classifying cells as totipotent, pluripotent, and multipotent^10^. Although pluripotency is a transient property of stem cells in vivo, regulation of signaling pathway activity involved in fate maintenance enables stem cells to remain in a pluripotent state for an extended period in vitro^85^. The pluripotent stage involves multiple stem cells in dynamically changing states, and the differentiation potential of each cell is gradually limited; therefore, the stem cell potential is determined on the basis of the developmental stage of the blastocyst from which the ESCs are derived. Due to the limited accessibility to human embryos^86^, studies using murine cells first elucidated the correlation between the origin, status, and differentiation capacity of PSCs. Two types of PSCs have been established from mouse blastocysts (Fig. 1c): ESCs^87,88^ and EpiSCs^53,54^. ESCs derived from the ICM of preimplanted mouse blastocysts were in a naive state and were capable of generating all three germ layers and primordial germ cells^87–89^; in contrast, EpiSCs isolated from postimplanted epiblasts were in the primed state and capable only of generating the three germ layers^53^. Although both ESCs and EpiSCs show the ability to form the three germ layers, these two types of stem cells are clearly distinguished by their developmental potential, as only ESCs, not EpiSCs, can efficiently generate chimeric progeny from injected blastocysts^53,54,90^. Differences in the ability of PSCs to generate chimeric animals are representative of stem cell potential in the in vivo counterparts of pre- or postimplantation blastocysts^91^. Various efforts have been made to elucidate the mechanism underlying the differences in pluripotency attributed to differences in PSC origin. In contrast to ESCs, which express a variety of pluripotency genes, including *Oct4*, *Nanog*, *Sox2*, *ESRRβ*, *Rex1*, *Klf2*, and *Klf4*, EpiSCs exhibit elevated expression levels of differentiation-inducing genes, such as *Otx2* and *Zic2*, and low levels of *Nanog*, *Rex1*, *Klf2*, or *Klf4*^54,89,92^. Studies designed to optimize in vitro culture conditions to maintain naive or primed PSCs have suggested that the endogenous activity of genes is regulated by signals transduced from outside a cell, such as fibroblast growth factor (FGF)^93^, bone morphogenic protein (BMP)^94^, transforming growth factor β (TGFβ)^95^, and WNT^96^. Therefore, early methods for culturing naive murine ESCs from mitotically inactive mouse embryonic fibroblasts and fetal bovine serum^97^ led to modulation of JAK-STAT3 pathway and NODAL signaling via the addition of leukemia inhibitory factor (LIF)^98^ and BMP^94^. Similarly, treatment with MEK/FGF/GSK3 inhibitors led to murine ESC maintenance in the naive state, even without LIF stimulation^96^. When FGF inhibition was released, cell proliferation was promoted while the naive state of the stem cells was maintained by a MEK-ERK-independent mechanism of endogenous FGF^93,99^. Alternatively, FGF was reported to induce the emergence of ESCs from the naive state. In addition, FGF together with activin A, abrogated the differentiation of murine ESCs into primordial germ cells^89^, whereas it promoted location-specific differentiation in an embryo; that is, it induced the acquisition of the anterior late-gastrula primitive streak cell phenotype by EpiSCs^100,101^. WNT signaling has also been found to function ambivalently with respect to the naive state of PSCs. For instance, the stabilization of nuclear β-catenin has been shown to enhance naive pluripotency by neutralizing the repressive activity of transcription factor 3 (TCF3) on nuclear target genes such as *Oct4*, *Nanog*, and *Sox2*^102–106^. The transcriptional activity of β-catenin on differentiation-associated target genes was diminished, thereby comporting with the increased membrane stability of cytoplasmic β-catenin rendered through complex formation with OCT4 and E-cadherin in cells in the naive state^107^. In contrast, by promoting mesodermal gene expression under differentiation-optimized conditions, nuclear β-catenin was shown to facilitate differentiation-related priming over naive pluripotency-promoting functions^108^.

Although hESCs are derived from blastocysts similar to those of mice, they differ from murine ESCs. For example, in contrast to murine ESCs, which require LIF for survival, hESCs derived from the ICM of blastocysts require FGF2 and TGF-β1. In addition, hESCs share hallmarks of murine EpiSCs, including partial expression of naive pluripotency markers, deposition of H3K27me3 at genetic loci required for development, lack of global hypomethylation, and an inactive X-chromosome in female ESC lines^51,109,110^. However, recent evidence has indicated that hESCs in the primed state resemble murine ESCs more closely than murine EpiSCs. Furthermore, hESCs maintain the expression of E-cadherin, which is highly expressed in naive murine ESCs, but not FGF5 or N-cadherin expression, although they are highly expressed in murine EpiSCs^51^. The level of expression of naive pluripotency factors such as *NANOG*, *PRDM14*, and *REX1* is relatively stable in hESCs, and ablation of NANOG and PRDM14 induced the differentiation of hESCs^111^. In addition, even when cultured with FGF2/activin A, the DNA methylation pattern of hESCs has been shown to be comparable to that of naive ESCs cultured in LIF/FBS not murine EpiSCs^112,113^.

To date, the distinction between naive and primed states remains unclear; however, clearly, the stem cell-specific regulation of intrinsic FGF and β-catenin signaling is precisely regulated at the early pluripotent naive stage.

## Control of gene expression in the pluripotent state

Cellular identity is driven by epigenomic, transcriptomic, and proteomic heterogeneity. Studies creating pluripotent cells by introducing the exogenous genes *OCT4*, *SOX2*, *KLF4*, and *MYC* have provided evidence for the key role played by the expression of specific genes in the regulation of stem cell fate^27,114^. More specifically, the aforementioned differences in the expression of symmetry disrupting and pluripotency genes are evident before the important division of PSCs into the three germ layers^66,87,115,116^. The regulation of autonomous molecular factors participating in the control mechanism of stem cell fate determines the homogeneous and naive states, with the function of these factors leading to cell-specific differences that lead to achieve heterogeneity and a primed cell state^117–119^.

In general, the regulation of gene expression required by cells is described by the central dogma^120^. Specifically, transcription, translation, posttranslational modifications or subsequent proteolytic processes precisely control the levels of molecular expression. In contrast to other processes that require the systematic organization and regulation of subsequent processes to control the level of a final molecular product, transcriptional regulation is an efficient process for regulating PSC fate because the molecular control mechanisms are immature^121–123^. Although the epigenome shapes stem cell hierarchies^124^, differential transcriptomes only partially explain protein abundance^121,122^. As hPSCs are characterized by completely open chromatin conditions and immature epigenetic systems, posttranscriptional control is thought to play a key role in the functional output of genetic programs (Fig. 1d)^125,126^. A zygote survives and functions through the support of molecular components passed down through gametes, which carried these traits before fertilization^127^. In particular, PSCs in the early stages of development undergo zygotic gene activation (ZGA), which allows precise regulation of selective expression of genes necessary for survival^128^. ZGA of hPSCs begins in the 2-cell stage and proceeds to the morula stage^129^, which is still characterized by immature chromatin reorganization, even in the ICM of a preimplantedblastocyte^130,131^. Although epigenetic control is thought to occur during this stage, epigenetic modifications are deposited during the heterogeneity acquisition process of the cell transition from a primed state to three germ layer development^31^. This timeline has been supported by studies showing de novo heterochromatin formation and X-chromosome inactivation in hPSCs after ICM formation^132–134^. In addition, as described above, WNT, FGF, or activin signaling regulates the transcription of molecular factors that affect global methylation through the activity of OCT4 and NANOG in the naive state^135,136^. This regulatory program requires additional mechanisms to compensate for coarse transcriptome level changes caused by immature transcriptional control prior to the completion of epigenome-driven transcriptional regulatory mechanisms^123^. Increasing data have suggested a role for posttranscriptional modifications in the early stages of stem cell development that enables the selective regulation of required functional outputs (i.e., proteome content) based on the global transcriptome.

### The epitranscriptome in development and stem cells

In addition to reported posttranscriptional modifications during oocyte development^137^, more than 100 chemical modifications in addition to typical posttranscriptional modifications, such as 5′-capping, polyadenylation, and splicing, have been identified^138^. Methylation is the most common enzyme-catalyzed modification, with N6-methyladenosine (m^6^A)^29^, N1-methyladenosine (m^1^A)^139^, pseudouridine (Ψ)^140^, and C5-methylcytosine (m^5^C)^141^ of RNA being reported. However, the role played by RNA m^6^A in posttranscriptional regulation during stem cell development is unknown.

### N6-methyladenosine (m^6^A)

m^6^A is the most prevalent modification of the 3′ untranslated regions (UTRs), long internal exons, intergenic regions, and 5′ UTRs in human mRNAs^30,142^. The functional m^6^A network is accurately orchestrated through a series of protein effector molecules, including “writers” for methylation, “erasers” for demethylation, and “readers” for interpretation of methylated mRNAs. A microRNA-guided protein complex consisting of Wilms tumor 1-associating protein (WTAP), methyltransferase-like protein 3 (METTL3), and methyltransferase-like protein 14 (METTL14) targets specific regions on mRNA, depositing a methyl group at the N6-position of adenosine^143–145^. Subsequent demethylation by eraser proteins, such as fat mass and obesity-associated protein (FTO) or human AlkB homolog H5 (ALKDH5), results in m^6^A being highly enriched in specific transcriptome sites^146,147^. The presence of eraser proteins that correct a methylation pattern suggests that m^6^A-associated posttranscriptional control might be a dynamic process that can quickly reflect environmental changes such as developmental progression and cellular stress. An m^6^A-loaded mRNA is recognized by RNA-binding protein YTH domain-containing families 1 (YTHDF1), 2 (YTHDF2), or 3 (YTHDF3) after being delivered to the cytoplasm through 5′ capping and polyadenylation^30,148,149^. Emerging evidence has suggested that YTHDF1, YTHDF2, and YTHDF3 target distinct mRNAs for cap-dependent translation, acceleration of mRNA decay, or promotion of translation and decay, respectively. In contrast to the prevailing model, recent studies have reported that all 3 YTHDFs act redundantly to mediate biological functions through specific m^6^A sites and shared target mRNAs^150^.

**Fig. 2:**
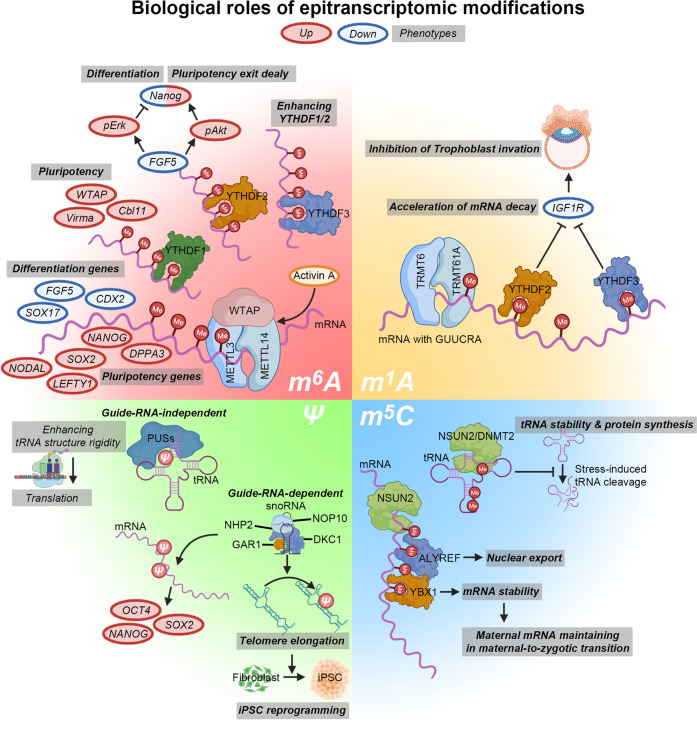
Various features of pluripotent stem cells are regulated by epitranscriptomic modifications. Molecular regulatory functions of epitranscriptomic modification m^6^A (red), m^1^A (yellow), pseudouridine (green), and m^5^C (blue) are associated with each biological phenotype in pluripotent stem cells.

Recent findings have also demonstrated the role played by the m^6^A modification in various molecular processes, such as translation efficiency, stability, localization, and splicing, which are involved in stem cell development and fate control (Fig. 2)^151–153^. Deficient METTL3 and METTL14 have been reported to inhibit the expression of pluripotent genes such as *SOX2*, *NANOG*, and *DPPA3* but to promote the expression of developmental regulators such as *FGF5*, *CDX2*, and *SOX17*^151^. Furthermore, deletion of *Mettl3* and *Mettl14* increased mRNA stability in a HuR- and miRNA-dependent manner^152^, a finding was further supported by a subsequent study showing that *ZC3H13* forms biochemical complexes with WTAP, VIRMA, and CBLL1^154^. This complex formation led to a decrease in global levels of the m^6^A modification and self-renewal ability, triggering the differentiation of murine ESCs. These results differed from those of a study reporting that *Mettl3*-knockout mice presented with embryonic lethality between embryonic days 3.5 and 6.5 when PSCs lost lineage differentiation potential^153^. In *Mettl3*-knockout murine ESCs, stabilized *FGF5* mRNA activated pErk and downregulated Nanog expression, FGF5-mediated concurrently coactivated pAkt to reestablish the expression of *Nanog*. The induction of differentiation and a concurrent delay in pluripotency loss might explain the discrepancy in these studies^155^. Ultimately, m^6^A loss causes confusion in determining whether cells maintain and advance in their pluripotent state and or undergo differentiation. Another study investigating the interactome of SMAD2/3 revealed that SMAD2/3 promoted m^6^A deposition on nuclear RNA by interacting with the METTL3-METTL14-WTAP complex in response to the activation of activin A/TGFβ signaling in primed hESCs^156^. Accumulation of the mRNA of NANOG, NODAL, and LEFTY1, which are activin A signaling targets, likely affects processes such as the transition from homogeneity to heterogeneity or from the naive to the primed state by dynamically reflecting extracellular signaling in hPSCs.

### Pseudouridine (ψ)

Pseudouridine is the most abundant modification on transfer RNAs (tRNAs) and ribosomal RNAs (rRNAs); it has also been also found on mRNAs, long noncoding RNAs (lncRNAs), and small nuclear RNAs (snRNAs)^157,158^. RNA is modified by pseudouridine synthases (PUSs) in a guide-RNA-independent manner^159^, affecting RNA structures by promoting base stacking interactions via a hydrogen bond donor to increase RNA backbone rigidity, thereby regulating the interaction of other biomolecules with RNAs^160^. Ultimately, pseudouridylation plays a role in increasing tRNAs stability and simultaneously alters translation termination, thereby regulating protein synthesis^161,162^. Interestingly, guide-RNA-dependent pseudouridylation is regulated by dyskerin (DKC1)^163^, which promotes the elongation of telomeres and increases the expression of OCT4 and SOX2^164^. However, mutations in DKC1 result in failed iPSC reprogramming^165^. In line with this outcome, DKC1 mutations have been found to be involved in the pathogenesis of human diseases characterized by short telomeres^163^. Moreover, deletion of *Dkc1* led to early embryonic lethality in a mouse model^166^. Although its association with the state of PSCs has not yet been clarified, pseudouridine might be related to stem cell development, maturation, and aging (Fig. 2).

### N1-methyladenosine (m^1^A)

In human cells, m^1^A is widely distributed on tRNAs^167^. The m^1^A modification is mediated by TRMT10C, TRMT61B, and TRMT6/61A methyltransferases and is associated with the maintenance of the structure, stability, and function of tRNAs in mitochondria or cytoplasm^162,168,169^. Less than 0.1% of mRNAs contain an m^1^A modification, which is generally located near the 5′ UTR close to a translation initiation site^139^. Although the role played by TRMT6/61A in depositing m^1^A modifications in cytoplasmic mRNAs carrying the GUUCRA tRNA-like motif is known, its contribution is minor^167^. Interestingly, similar to the m^6^A modification, the m^1^A modification involves a dynamic and reversible process. m^1^A demethylation is mediated by ALKBH1 and ALKBH3, and m^6^A and m^1^A share a reader protein^170,171^. YTHDF2 and YTHDF3 are known to contribute to the interpretation of m^1^A, causing the rapid decay of modified mRNAs^172,173^. Recent studies have suggested that YTHDF3 promotes the degradation of *IGF1R* mRNA, leading to the inhibition of trophoblast invasion in trophoblast-associated pregnancy disorders^173^. Although neither the biological role nor the molecular mechanism of m^1^A has been established to date, it is thought to be involved in the pathophysiology of stem cells because of the similarity in the dynamics and shared molecular machineries of the m^1^A and m^6^A modifications (Fig. 2).

### C5-methylcytosine (m^5^C)

Similar to m^6^A, the m^5^C modification is reversible and controlled by methyltransferases (writers: NSUNs, TRDMT1, and DNMT2)^174^ and demethylases (erasers: TETs and ALKBH1)^175–177^. The molecular function of m^5^C is mediated by reader proteins (ALYREF and YBX1)^178^. Although the m^5^C modification is highly enriched on tRNAs and rRNAs, it reportedly plays a role in mRNA export^178^, RNA stability maintenance^178^, and translation^179^. In zebrafish, deletion of maternal Ybx1 enhanced translation and triggered the unfolded protein response, resulting in oogenesis and embryogenesis defects^180^. In addition, Ybx1 has been shown to play a role in maintaining maternal m^5^C-containing mRNAs during the maternal-to-zygotic transition (MZT) during initial embryogenesis^181^. These mRNAs have been reported to facilitate the MZT in early zebrafish development. A pathological association between m^5^C and the activation of JAK-STAT signaling and dysregulation of tRNAs has been reported in various cancers and neurodegenerative disorders in humans^178,182^. Although the role played by the m^5^C modification in the regulation of early human stem cell properties has not been elucidated, further research into the function of m^5^C in health and disease is warranted because of its effect on early zebrafish embryogenesis and importance of TET proteins in early stem cell development (Fig. 2).

## Conclusions and perspectives

Although somatic cells control chromatin accessibility to transcription machineries through their well-organized heterochromatin structure and epigenetic control, hPSCs accommodate the uncontrolled accessibility of transcriptional machineries to euchromatin throughout the nucleoplasm. However, PSCs strictly control the levels of expression of certain proteins, which are the functional end products of strictly controlled gene expression. More specifically, the molecules that determine the differentiation potential and developmental stage of PSCs are controlled at the epitranscriptomic level. Epitranscriptomic regulation, which controls the stability and translational efficiency of transcribed mRNAs, overcomes unfavorable conditions faced by PSCs. In particular, m^6^A is the most prevalent epitranscriptomic modification of mRNAs and is involved in the regulation of major signaling pathways in hPSCs. However, a number of studies have shown that mRNA modifications in addition to m^6^A were correlated with early developmental changes in animal models or modulated by molecular mechanisms during early developmental changes in PSCs. Therefore, future studies should be directed to investigating the complex epitranscriptomic gene regulatory system, which involves the mutual interaction of multiple types of RNA modifications, as well as the molecular mechanism and biological implications of certain types of RNA modifications.

## References

[1] Fuchs E, Segre JA (2000). Stem cells: a new lease on life. Cell.

[2] Winickoff DE, Saha K, Graff GD (2009). Opening stem cell research and development: a policy proposal for the management of data, intellectual property, and ethics. Yale J. Health Policy Law Ethics.

[3] Stent GS (1985). The role of cell lineage in development. Philos. Trans. R. Soc. Lond. B Biol. Sci..

[4] Wobus AM, Boheler KR (2005). Embryonic stem cells: prospects for developmental biology and cell therapy. Physiol. Rev..

[5] Yun W, Kim YJ, Lee G (2022). Direct conversion to achieve glial cell fates: oligodendrocytes and Schwann cells. Int. J. Stem Cells.

[6] Lee G (2009). Modelling pathogenesis and treatment of familial dysautonomia using patient-specific iPSCs. Nature.

[7] Lee G (2012). Large-scale screening using familial dysautonomia induced pluripotent stem cells identifies compounds that rescue IKBKAP expression. Nat. Biotechnol..

[8] Kim YJ (2014). Generation of multipotent induced neural crest by direct reprogramming of human postnatal fibroblasts with a single transcription factor. Cell Stem Cell.

[9] Larsen, W. J., Sherman, L. S., Potter, S. S. & Scott, W. J. *Human Embryology*. 3rd edn, 20 (Churchill Livingstone, 2001).

[10] Mitalipov S, Wolf D (2009). Totipotency, pluripotency and nuclear reprogramming. Adv. Biochem. Eng. Biotechnol..

[11] Thomson JA (1998). Embryonic stem cell lines derived from human blastocysts. Science.

[12] Popovic M, Azpiroz F, Chuva de Sousa Lopes SM (2021). Engineered models of the human embryo. Nat. Biotechnol..

[13] Choi IY, Lim HT, Che YH, Lee G, Kim YJ (2021). Inhibition of the combinatorial signaling of transforming growth factor-beta and NOTCH promotes myotube formation of human pluripotent stem cell-derived skeletal muscle progenitor cells. Cells.

[14] Lyoo KS (2022). Direct neuronal infection of SARS-CoV-2 reveals cellular and molecular pathology of chemosensory impairment of COVID-19 patients. Emerg. Microbes Infect..

[15] Weinberger L, Ayyash M, Novershtern N, Hanna JH (2016). Dynamic stem cell states: naive to primed pluripotency in rodents and humans. Nat. Rev. Mol. Cell Biol..

[16] Morris SA (2010). Origin and formation of the first two distinct cell types of the inner cell mass in the mouse embryo. Proc. Natl Acad. Sci. USA.

[17] Rebuzzini P, Zuccotti M, Garagna S (2021). Building pluripotency identity in the early embryo and derived stem cells. Cells.

[18] Minn KT (2020). High-resolution transcriptional and morphogenetic profiling of cells from micropatterned human ESC gastruloid cultures. Elife.

[19] Avila-Gonzalez D (2021). Unraveling the spatiotemporal human pluripotency in embryonic development. Front. Cell Dev. Biol..

[20] Gardner RL (1983). Origin and differentiation of extraembryonic tissues in the mouse. Int. Rev. Exp. Pathol..

[21] Nichols J, Gardner RL (1984). Heterogeneous differentiation of external cells in individual isolated early mouse inner cell masses in culture. J. Embryol. Exp. Morphol..

[22] Lander AD (2012). What does the concept of the stem cell niche really mean today?. BMC Biol..

[23] Peerani R (2007). Niche-mediated control of human embryonic stem cell self-renewal and differentiation. EMBO J..

[24] Schoger E, Argyriou L, Zimmermann WH, Cyganek L, Zelarayan LC (2020). Generation of homozygous CRISPRa human induced pluripotent stem cell (hiPSC) lines for sustained endogenous gene activation. Stem Cell Res..

[25] Bhattacharya B, Puri S, Puri RK (2009). A review of gene expression profiling of human embryonic stem cell lines and their differentiated progeny. Curr. Stem Cell Res. Ther..

[26] Messmer T (2019). Transcriptional heterogeneity in naive and primed human pluripotent stem cells at single-cell resolution. Cell Rep..

[27] Takahashi K (2007). Induction of pluripotent stem cells from adult human fibroblasts by defined factors. Cell.

[28] Lunyak VV, Rosenfeld MG (2008). Epigenetic regulation of stem cell fate. Hum. Mol. Genet..

[29] Desrosiers R, Friderici K, Rottman F (1974). Identification of methylated nucleosides in messenger RNA from Novikoff hepatoma cells. Proc. Natl Acad. Sci. USA.

[30] Wang X (2014). N6-methyladenosine-dependent regulation of messenger RNA stability. Nature.

[31] Meshorer E, Misteli T (2006). Chromatin in pluripotent embryonic stem cells and differentiation. Nat. Rev. Mol. Cell Biol..

[32] Gupta S, Santoro R (2020). Regulation and Roles of the Nucleolus in Embryonic Stem Cells: From Ribosome Biogenesis to Genome Organization. Stem Cell Rep..

[33] Shpiz A (2015). Human embryonic stem cells carrying an unbalanced translocation demonstrate impaired differentiation into trophoblasts: an in vitro model of human implantation failure. Mol. Hum. Reprod..

[34] Yung S (2011). Large-scale transcriptional profiling and functional assays reveal important roles for Rho-GTPase signalling and SCL during haematopoietic differentiation of human embryonic stem cells. Hum. Mol. Genet..

[35] Petropoulos S (2016). Single-cell RNA-Seq reveals lineage and X chromosome dynamics in human preimplantation embryos. Cell.

[36] Shahbazi MN (2016). Self-organization of the human embryo in the absence of maternal tissues. Nat. Cell Biol..

[37] Xiang L (2020). A developmental landscape of 3D-cultured human pre-gastrulation embryos. Nature.

[38] Wamaitha SE, Niakan KK (2018). Human pre-gastrulation development. Curr. Top. Dev. Biol..

[39] Stirparo GG (2018). Correction: Integrated analysis of single-cell embryo data yields a unified transcriptome signature for the human pre-implantation epiblast. Development.

[40] Mole MA, Weberling A, Zernicka-Goetz M (2020). Comparative analysis of human and mouse development: from zygote to pre-gastrulation. Curr. Top. Dev. Biol..

[41] Tarrade A (2001). Characterization of human villous and extravillous trophoblasts isolated from first trimester placenta. Lab. Investig..

[42] Sheng G (2015). Epiblast morphogenesis before gastrulation. Dev. Biol..

[43] Sasaki K (2016). The germ cell fate of cynomolgus monkeys is specified in the nascent amnion. Dev. Cell.

[44] Chen D (2019). Human primordial germ cells are specified from lineage-primed progenitors. Cell Rep..

[45] Kobayashi T, Surani MA (2018). On the origin of the human germline. Development.

[46] Nakamura T (2016). A developmental coordinate of pluripotency among mice, monkeys and humans. Nature.

[47] Shahbazi MN, Siggia ED, Zernicka-Goetz M (2019). Self-organization of stem cells into embryos: a window on early mammalian development. Science.

[48] Strelchenko N, Verlinsky O, Kukharenko V, Verlinsky Y (2004). Morula-derived human embryonic stem cells. Reprod. Biomed. Online.

[49] Bongso A, Fong CY, Ng SC, Ratnam S (1994). Isolation and culture of inner cell mass cells from human blastocysts. Hum. Reprod..

[50] Io S (2021). Capturing human trophoblast development with naive pluripotent stem cells in vitro. Cell Stem Cell.

[51] Gafni O (2013). Derivation of novel human ground state naive pluripotent stem cells. Nature.

[52] Theunissen TW (2014). Systematic identification of culture conditions for induction and maintenance of naive human pluripotency. Cell Stem Cell.

[53] Brons IG (2007). Derivation of pluripotent epiblast stem cells from mammalian embryos. Nature.

[54] Tesar PJ (2007). New cell lines from mouse epiblast share defining features with human embryonic stem cells. Nature.

[55] Huang Y, Osorno R, Tsakiridis A, Wilson V (2012). In Vivo differentiation potential of epiblast stem cells revealed by chimeric embryo formation. Cell Rep..

[56] Hayashi K, Saitou M (2014). Perspectives of germ cell development in vitro in mammals. Anim. Sci. J..

[57] Fang R (2014). Generation of naive induced pluripotent stem cells from rhesus monkey fibroblasts. Cell Stem Cell.

[58] Honda A (2013). Naive-like conversion overcomes the limited differentiation capacity of induced pluripotent stem cells. J. Biol. Chem..

[59] Jehle H (1970). Bilateral symmetry in morphogenesis of embryos. Proc. Natl Acad. Sci. USA.

[60] Tam PP, Williams EA, Chan WY (1993). Gastrulation in the mouse embryo: ultrastructural and molecular aspects of germ layer morphogenesis. Microsc. Res. Tech..

[61] Tam PP, Behringer RR (1997). Mouse gastrulation: the formation of a mammalian body plan. Mech. Dev..

[62] Arnold SJ, Robertson EJ (2009). Making a commitment: cell lineage allocation and axis patterning in the early mouse embryo. Nat. Rev. Mol. Cell Biol..

[63] Tam PP, Loebel DA (2007). Gene function in mouse embryogenesis: get set for gastrulation. Nat. Rev. Genet..

[64] Casser E (2017). Totipotency segregates between the sister blastomeres of two-cell stage mouse embryos. Sci. Rep..

[65] Biase FH, Cao X, Zhong S (2014). Cell fate inclination within 2-cell and 4-cell mouse embryos revealed by single-cell RNA sequencing. Genome Res..

[66] Shi J (2015). Dynamic transcriptional symmetry-breaking in pre-implantation mammalian embryo development revealed by single-cell RNA-seq. Development.

[67] Zdravkovic T (2015). Human stem cells from single blastomeres reveal pathways of embryonic or trophoblast fate specification. Development.

[68] Tsunoda Y, McLaren A (1983). Effect of various procedures on the viability of mouse embryos containing half the normal number of blastomeres. J. Reprod. Fertil..

[69] Morris SA, Guo Y, Zernicka-Goetz M (2012). Developmental plasticity is bound by pluripotency and the Fgf and Wnt signaling pathways. Cell Rep..

[70] Papaioannou VE, Ebert KM (1995). Mouse half embryos: viability and allocation of cells in the blastocyst. Dev. Dyn..

[71] Katayama M, Ellersieck MR, Roberts RM (2010). Development of monozygotic twin mouse embryos from the time of blastomere separation at the two-cell stage to blastocyst. Biol. Reprod..

[72] Goolam M (2016). Heterogeneity in Oct4 and Sox2 targets biases cell fate 4-CEll Mouse Embryos. Cell Fate 4-Cell Mouse Embryos. Cell.

[73] Burton A (2013). Single-cell profiling of epigenetic modifiers identifies PRDM14 as an inducer of cell fate in the mammalian embryo. Cell Rep..

[74] White MD (2016). Long-lived binding of Sox2 to DNA predicts cell fate four-cell mouse embryo. Cell.

[75] Torres-Padilla ME, Parfitt DE, Kouzarides T, Zernicka-Goetz M (2007). Histone arginine methylation regulates pluripotency in the early mouse embryo. Nature.

[76] Kuzmichev AN (2012). Sox2 acts through Sox21 to regulate transcription in pluripotent and differentiated cells. Curr. Biol..

[77] Strumpf D (2005). Cdx2 is required for correct cell fate specification and differentiation of trophectoderm in the mouse blastocyst. Development.

[78] Posfai E (2017). Position- and Hippo signaling-dependent plasticity during lineage segregation in the early mouse embryo. Elife.

[79] Jedrusik A (2008). Role of Cdx2 and cell polarity in cell allocation and specification of trophectoderm and inner cell mass in the mouse embryo. Genes Dev..

[80] Skamagki M, Wicher KB, Jedrusik A, Ganguly S, Zernicka-Goetz M (2013). Asymmetric localization of Cdx2 mRNA during the first cell-fate decision in early mouse development. Cell Rep..

[81] Dietrich JE, Hiiragi T (2007). Stochastic patterning in the mouse pre-implantation embryo. Development.

[82] Wennekamp S, Hiiragi T (2012). Stochastic processes in the development of pluripotency in vivo. Biotechnol. J..

[83] Tabansky I (2013). Developmental bias in cleavage-stage mouse blastomeres. Curr. Biol..

[84] Plachta N, Bollenbach T, Pease S, Fraser SE, Pantazis P (2011). Oct4 kinetics predict cell lineage patterning in the early mammalian embryo. Nat. Cell Biol..

[85] Nichols J, Smith A (2012). Pluripotency in the embryo and in culture. Cold Spring Harb. Perspect. Biol..

[86] Lovell-Badge R (2008). The regulation of human embryo and stem-cell research in the United Kingdom. Nat. Rev. Mol. Cell Biol..

[87] Evans MJ, Kaufman MH (1981). Establishment in culture of pluripotential cells from mouse embryos. Nature.

[88] Martin GR (1981). Isolation of a pluripotent cell line from early mouse embryos cultured in medium conditioned by teratocarcinoma stem cells. Proc. Natl Acad. Sci. USA.

[89] Hayashi K, Ohta H, Kurimoto K, Aramaki S, Saitou M (2011). Reconstitution of the mouse germ cell specification pathway in culture by pluripotent stem cells. Cell.

[90] Bradley A, Evans M, Kaufman MH, Robertson E (1984). Formation of germ-line chimaeras from embryo-derived teratocarcinoma cell lines. Nature.

[91] Brook FA, Gardner RL (1997). The origin and efficient derivation of embryonic stem cells in the mouse. Proc. Natl Acad. Sci. USA.

[92] Hackett JA, Surani MA (2014). Regulatory principles of pluripotency: from the ground state up. Cell Stem Cell.

[93] Chen H (2015). Erk signaling is indispensable for genomic stability and self-renewal of mouse embryonic stem cells. Proc. Natl Acad. Sci. USA.

[94] Ying QL, Nichols J, Chambers I, Smith A (2003). BMP induction of Id proteins suppresses differentiation and sustains embryonic stem cell self-renewal in collaboration with STAT3. Cell.

[95] Kattman SJ (2011). Stage-specific optimization of activin/nodal and BMP signaling promotes cardiac differentiation of mouse and human pluripotent stem cell lines. Cell Stem Cell.

[96] Ying QL (2008). The ground state of embryonic stem cell self-renewal. Nature.

[97] Martin GR, Evans MJ (1975). Differentiation of clonal lines of teratocarcinoma cells: formation of embryoid bodies in vitro. Proc. Natl Acad. Sci. USA.

[98] Williams RL (1988). Myeloid leukaemia inhibitory factor maintains the developmental potential of embryonic stem cells. Nature.

[99] Buehr M (2008). Capture of authentic embryonic stem cells from rat blastocysts. Cell.

[100] Kojima Y (2014). The transcriptional and functional properties of mouse epiblast stem cells resemble the anterior primitive streak. Cell Stem Cell.

[101] Han DW (2010). Epiblast stem cell subpopulations represent mouse embryos of distinct pregastrulation stages. Cell.

[102] Marson A (2008). Wnt signaling promotes reprogramming of somatic cells to pluripotency. Cell Stem Cell.

[103] Cole MF, Johnstone SE, Newman JJ, Kagey MH, Young RA (2008). Tcf3 is an integral component of the core regulatory circuitry of embryonic stem cells. Genes Dev..

[104] Tam WL (2008). T-cell factor 3 regulates embryonic stem cell pluripotency and self-renewal by the transcriptional control of multiple lineage pathways. Stem Cells.

[105] Yi F, Pereira L, Merrill BJ (2008). Tcf3 functions as a steady-state limiter of transcriptional programs of mouse embryonic stem cell self-renewal. Stem Cells.

[106] Kim S (2020). The distinct role of Tcfs and Lef1 in the self-renewal or differentiation of mouse embryonic stem cells. Int. J. Stem Cells.

[107] Faunes F (2013). A membrane-associated beta-catenin/Oct4 complex correlates with ground-state pluripotency in mouse embryonic stem cells. Development.

[108] Chen Y, Blair K, Smith A (2013). Robust self-renewal of rat embryonic stem cells requires fine-tuning of glycogen synthase kinase-3 inhibition. Stem Cell Rep..

[109] Smith ZD (2014). DNA methylation dynamics of the human preimplantation embryo. Nature.

[110] Mekhoubad S (2012). Erosion of dosage compensation impacts human iPSC disease modeling. Cell Stem Cell.

[111] Chia NY (2010). A genome-wide RNAi screen reveals determinants of human embryonic stem cell identity. Nature.

[112] Hackett JA (2013). Synergistic mechanisms of DNA demethylation during transition to ground-state pluripotency. Stem Cell Rep..

[113] Shipony Z (2014). Dynamic and static maintenance of epigenetic memory in pluripotent and somatic cells. Nature.

[114] Park IH (2008). Reprogramming of human somatic cells to pluripotency with defined factors. Nature.

[115] Bayerl J (2021). Principles of signaling pathway modulation for enhancing human naive pluripotency induction. Cell Stem Cell.

[116] Taelman J (2019). WNT inhibition and increased FGF signaling promotes derivation of less heterogeneous primed human embryonic stem cells, compatible with differentiation. Stem Cells Dev..

[117] Linneberg-Agerholm M (2019). Naive human pluripotent stem cells respond to Wnt, Nodal and LIF signalling to produce expandable naive extra-embryonic endoderm. Development.

[118] Kurek D (2015). Endogenous WNT signals mediate BMP-induced and spontaneous differentiation of epiblast stem cells and human embryonic stem cells. Stem Cell Rep..

[119] Wu J (2015). An alternative pluripotent state confers interspecies chimaeric competency. Nature.

[120] Nirenberg M (2004). Historical review: deciphering the genetic code-a personal account. Trends Biochem. Sci..

[121] Player A (2006). Comparisons between transcriptional regulation and RNA expression in human embryonic stem cell lines. Stem Cells Dev..

[122] Fathi A (2009). Comparative proteome and transcriptome analyses of embryonic stem cells during embryoid body-based differentiation. Proteomics.

[123] Efroni S (2008). Global transcription in pluripotent embryonic stem cells. Cell Stem Cell.

[124] Moris N, Pina C, Arias AM (2016). Transition states and cell fate decisions in epigenetic landscapes. Nat. Rev. Genet..

[125] Chen Q, Hu G (2017). Post-transcriptional regulation of the pluripotent state. Curr. Opin. Genet. Dev..

[126] van den Berg PR, Budnik B, Slavov N, Semrau S. Dynamic post-transcriptional regulation during embryonic stem cell differentiation. Preprint at *biorxiv*10.1101/123497 (2017).

[127] Zhang C, Wang M, Li Y, Zhang Y (2022). Profiling and functional characterization of maternal mRNA translation during mouse maternal-to-zygotic transition. Sci. Adv..

[128] Jukam D, Shariati SAM, Skotheim JM (2017). Zygotic genome activation in vertebrates. Dev. Cell.

[129] Sha QQ (2020). Dynamics and clinical relevance of maternal mRNA clearance during the oocyte-to-embryo transition in humans. Nat. Commun..

[130] Burton A, Torres-Padilla ME (2014). Chromatin dynamics in the regulation of cell fate allocation during early embryogenesis. Nat. Rev. Mol. Cell Biol..

[131] Aoto T, Saitoh N, Ichimura T, Niwa H, Nakao M (2006). Nuclear and chromatin reorganization in the MHC-Oct3/4 locus at developmental phases of embryonic stem cell differentiation. Dev. Biol..

[132] Lengner CJ (2010). Derivation of pre-X inactivation human embryonic stem cells under physiological oxygen concentrations. Cell.

[133] Kresoja-Rakic J, Santoro R (2019). Nucleolus and rRNA gene chromatin in early embryo development. Trends Genet..

[134] Berdasco M, Esteller M (2011). DNA methylation in stem cell renewal and multipotency. Stem Cell Res. Ther..

[135] Gao L (2018). Chromatin accessibility landscape in human early embryos and its association with evolution. Cell.

[136] Wu J (2018). Chromatin analysis in human early development reveals epigenetic transition during ZGA. Nature.

[137] Ozban N, Tandler J, Sirlin JL (1964). Methylation of nucleolar RNA during development of the amphibian ooecyte. J. Embryol. Exp. Morphol..

[138] Bentley DL (2014). Coupling mRNA processing with transcription in time and space. Nat. Rev. Genet..

[139] Dominissini D (2016). The dynamic N(1)-methyladenosine methylome in eukaryotic messenger RNA. Nature.

[140] Carlile TM (2014). Pseudouridine profiling reveals regulated mRNA pseudouridylation in yeast and human cells. Nature.

[141] Khoddami V, Cairns BR (2013). Identification of direct targets and modified bases of RNA cytosine methyltransferases. Nat. Biotechnol..

[142] Yue Y, Liu J, He C (2015). RNA N6-methyladenosine methylation in post-transcriptional gene expression regulation. Genes Dev..

[143] Liu J (2014). A METTL3-METTL14 complex mediates mammalian nuclear RNA N6-adenosine methylation. Nat. Chem. Biol..

[144] Ping XL (2014). Mammalian WTAP is a regulatory subunit of the RNA N6-methyladenosine methyltransferase. Cell Res..

[145] Chen T (2015). m(6)A RNA methylation is regulated by microRNAs and promotes reprogramming to pluripotency. Cell Stem Cell.

[146] Jia G (2011). N6-methyladenosine in nuclear RNA is a major substrate of the obesity-associated FTO. Nat. Chem. Biol..

[147] Zheng G (2013). ALKBH5 is a mammalian RNA demethylase that impacts RNA metabolism and mouse fertility. Mol. Cell.

[148] Wang X (2015). N(6)-methyladenosine modulates messenger RNA translation efficiency. Cell.

[149] Shi H (2017). YTHDF3 facilitates translation and decay of N(6)-methyladenosine-modified RNA. Cell Res..

[150] Zaccara S, Jaffrey SR (2020). A unified model for the function of YTHDF proteins in regulating m(6)A-modified mRNA. Cell.

[151] Batista PJ (2014). m(6)A RNA modification controls cell fate transition in mammalian embryonic stem cells. Cell Stem Cell.

[152] Wang Y (2014). N6-methyladenosine modification destabilizes developmental regulators in embryonic stem cells. Nat. Cell Biol..

[153] Geula S (2015). Stem cells. m6A mRNA methylation facilitates resolution of naive pluripotency toward differentiation. Science.

[154] Weng H (2018). METTL14 inhibits hematopoietic stem/progenitor differentiation and promotes leukemogenesis via mRNA m(6)A modification. Cell Stem Cell.

[155] Jin KX (2021). N6-methyladenosine (m(6)A) depletion regulates pluripotency exit by activating signaling pathways in embryonic stem cells. Proc. Natl Acad. Sci. USA.

[156] Bertero A (2018). The SMAD2/3 interactome reveals that TGFbeta controls m(6)A mRNA methylation in pluripotency. Nature.

[157] Li X (2015). Chemical pulldown reveals dynamic pseudouridylation of the mammalian transcriptome. Nat. Chem. Biol..

[158] Nachtergaele S, He C (2018). Chemical modifications in the life of an mRNA transcript. Annu. Rev. Genet..

[159] Hamma T, Ferre-D’Amare AR (2006). Pseudouridine synthases. Chem. Biol..

[160] Charette M, Gray MW (2000). Pseudouridine in RNA: what, where, how, and why. IUBMB Life.

[161] De Zoysa MD, Yu YT (2017). Posttranscriptional RNA pseudouridylation. Enzymes.

[162] Roundtree IA, Evans ME, Pan T, He C (2017). Dynamic RNA modifications in gene expression regulation. Cell.

[163] Batista LF (2011). Telomere shortening and loss of self-renewal in dyskeratosis congenita induced pluripotent stem cells. Nature.

[164] Fong YW, Ho JJ, Inouye C, Tjian R (2014). The dyskerin ribonucleoprotein complex as an OCT4/SOX2 coactivator in embryonic stem cells. Elife.

[165] Agarwal S (2010). Telomere elongation in induced pluripotent stem cells from dyskeratosis congenita patients. Nature.

[166] He J (2002). Targeted disruption of Dkc1, the gene mutated in X-linked dyskeratosis congenita, causes embryonic lethality in mice. Oncogene.

[167] Li X (2017). Base-resolution mapping reveals distinct m(1)A methylome in nuclear- and mitochondrial-encoded transcripts. Mol. Cell.

[168] Li X (2016). Transcriptome-wide mapping reveals reversible and dynamic N(1)-methyladenosine methylome. Nat. Chem. Biol..

[169] Macari F (2016). TRM6/61 connects PKCalpha with translational control through tRNAi(Met) stabilization: impact on tumorigenesis. Oncogene.

[170] Liu F (2016). ALKBH1-Mediated tRNA demethylation regulates translation. Cell.

[171] Chen Z (2019). Transfer RNA demethylase ALKBH3 promotes cancer progression via induction of tRNA-derived small RNAs. Nucleic Acids Res..

[172] Seo KW, Kleiner RE (2020). YTHDF2 recognition of N(1)-methyladenosine (m(1)A)-modified RNA is associated with transcript destabilization. ACS Chem. Biol..

[173] Zheng Q (2020). Cytoplasmic m(1)A reader YTHDF3 inhibits trophoblast invasion by downregulation of m(1)A-methylated IGF1R. Cell Discov..

[174] Chen YS, Yang WL, Zhao YL, Yang YG (2021). Dynamic transcriptomic m(5) C and its regulatory role in RNA processing. Wiley Interdiscip. Rev. RNA.

[175] Fu L (2014). Tet-mediated formation of 5-hydroxymethylcytosine in RNA. J. Am. Chem. Soc..

[176] Shen Q (2018). Tet2 promotes pathogen infection-induced myelopoiesis through mRNA oxidation. Nature.

[177] Kawarada L (2017). ALKBH1 is an RNA dioxygenase responsible for cytoplasmic and mitochondrial tRNA modifications. Nucleic Acids Res..

[178] Chen X (2019). 5-methylcytosine promotes pathogenesis of bladder cancer through stabilizing mRNAs. Nat. Cell Biol..

[179] Tuorto F (2012). RNA cytosine methylation by Dnmt2 and NSun2 promotes tRNA stability and protein synthesis. Nat. Struct. Mol. Biol..

[180] Sun J, Yan L, Shen W, Meng A (2018). Maternal Ybx1 safeguards zebrafish oocyte maturation and maternal-to-zygotic transition by repressing global translation. Development.

[181] Yang Y (2019). RNA 5-methylcytosine facilitates the maternal-to-zygotic transition by preventing maternal mRNA Decay. Mol. Cell.

[182] Blanco S (2014). Aberrant methylation of tRNAs links cellular stress to neuro-developmental disorders. EMBO J..

